# A pre-protective objective in mining females social contents for identification of early signs of depression using soft computing deep framework

**DOI:** 10.1038/s41598-023-40607-6

**Published:** 2023-09-09

**Authors:** Hanen Karamti, Abeer M. Mahmoud

**Affiliations:** 1 Computer Sciences Department, College of Computer and Information Sciences, Princess Nourah bint Abdulrahman University, Riyadh, 84428 Kingdom of Saudi Arabia; 2 Computer Science Department, Faculty of Computer and Information Sciences, Ain Shams University, Cairo, Egypt

**Keywords:** Health care, Medical research, Signs and symptoms

## Abstract

Currently, a noteworthy volume of information is available and shared every day through participation and communication of individuals on social media. These enormous contents with the right exploit and research leads to valuable discoveries. In this study, a deep framework of learning accurate detection of women’s depression is proposed. It is beneficially guided by social media content of individual posts and tweets and an essential support from psycho-linguistic for providing the indicator depression signs vocabulary that creates the embedding words necessary for building the applied approach. The presented model is validated using dual datasets extracted from Twitter: the first dataset is general data formed by 700 women from different countries; the second contains only 80 women from KSA. A third benchmark dataset CLPsych 2015 is used for comparative analysis purposes. The model proved its performance on the three datasets and the obtained and reported in this paper results shows its effectiveness.

## Introduction

Mining social media with main objectives of valuable discoveries is an emerging technology today. Social media has become the main to share information (text, audio, image, and video) on social networking sites, to communicate with our family, friends, and colleagues. We consume a large portion of time per day to view, share, comment, and like a massive collection of images or text on social media. Accordingly, through social media, we can express our emotions and talk about our events, diseases, institutions, etc. In addition, these online communities provides a range of different helping hands like (1) real experts in the domain searching for publicity and being famous through introducing free contents and/or (2) individuals with experiences in similar situations, evaluating some treatment options, and sources. These influence researchers in public health, to give their attention and efforts to predict some emotional diseases from this huge available interactive rich data. Focusing on depression as a major health problem recently and main cause for many other regarded as unwelcome issues in public health^1–3^ especially for women that face more stress than men mostly in her daily activates; the authors in this study were motivated by studying and building an aided system for the detection and analysis of women’s depression.

There are many reasons to choose Twitter as the source of data for the computational experiments of this work. Among these reasons, we can cite the big mass of tweets that Twitter have^1,4^, its generated rates reach more than 250 billion per year, and about 350,000 tweets per minute with unlimited increase every second. In addition, the texts on Twitter are constrained to a maximum number of words (140 characters per tweet) compared with other text sources which facilitates the mining process with different probabilistic and statistical methods or even recent deep models. Also, Twitter has rich contents on healthcare information in general and for gaining consciousness of depression for women in recent years in specific^5^.

The feature vectorization is a vital phase in text classification because it is the key contributor to reach accuracy. Actually, noticeable literature on the detection of depression using social media networks exists^6^. Most of the researchers followed two main steps in their work; first they identified the important features as an indicator of depression using different methods for text classification then they constructed heir predictive models^7^. This formulation presents a huge challenge for them to reach valuable discoveries and in rapid performance with the huge amount of generated data.. Motivated by the previous problem statement and following the mentioned literature directions in building a model for detection of women depression from social content, we propose in the present paper the combination of word embedding algorithm with a proposed deep architecture. The word embedding focuses on preparing features and formulating a suitable input for training the proposed deep learning model efficiently. The main contribution of this study is developing a new merged framework based on deep learning and word embedding algorithm. The developed framework focuses to discover the pattern of women tweets identify weather there is depression signs or not in their contents. This indirectly contributes in many issues relates to public health and early handling of these issues also has great impact of families and societies. The authors in addition validated their results on three type of datasets extracted from Twitter: (1) the first dataset is general data formed by 700 women from different countries; (2) the second dataset contains only 80 women from KSA. A third benchmark dataset CLPsych 2015 is used for comparative analysis purposes.

In the following section, some recent related works that attempt concepts of feature extraction based on the sentiment analysis, however with different algorithms and parameters setting of the training models^8,9^. Differently, their goal was to understand the relationship between users. They also exhibit how graphical expressions and their connection with other social accounts can influence the performance. Their experiments showed a strong relationship between emotions and the behaviors of users. In this study, we set a proposition that the individual expresses his/her feeling through publishing on Twitter with followers. This certainly provides relations between his/her behavior and inner mode condition. Then, from those emotions, we extract and demonstrate such relation that can be used for early detection of women’s depression.

The paper follows as: section “Related work” surveys the related social based mining for depression signs and symptoms in general. Section “Applied method and detailed pre-processing” describes the proposed methodology from corpus construction to model development. Section “Experimental and discussion” displays the obtained results. Section “Conclusion and future work” summarizes the findings and suggests future directions.

## Related work

Social media is a huge source of data about behaviors and emotions for events in peoples’ lives. This can take the form of posts, images commentary, etc. Many previous attempts tried to estimate how individuals’ appropriate social media and benefit from the health advises relative to its rapid responses and huge participants. In this section, several attempts have been presented to detect the main recent concern of suicide (depression)^8,10^. The related methods were categorized to statistic based or machine learning based models.

Several authors preferred to use statistical techniques to build their model for depression detection; for example Ref.^11^ examined the depression disease in Saudi Arabia, exactly in Buraydah city, in teenagers from 15 to 19 years during the summer of 2014. The entire “teenagers” were active in their Twitter accounts, they also used social media daily, they shared their mental health (depression), and social activities. The data contains 80 females and the proposed model to detect depression was a statistical model. The signs of depression were scaled based on medical scoring method and the results indicated that 35% of individuals were depressed while 60% were normal.

Other authors used the machine learning techniques to create their models, where they employed shallow techniques like SVM (Support Vector Machine)^12^, K-means^13^, and neural network^8^ or deep learning techniques like CNN^14^, DNN^15^ and RNN (Recurrent Neural Network)^16^. Authors in^17^ used the SVM as a supervised method and studied the new mothers’ risk of postpartum depression detected from Facebook contents. The researchers in^10^ choose the Facebook as a source of data to detect depression and developed the following classifiers: (1) SVM, (2) Decision Tree, and (3) k-Nearest Neighbor (kNN). They used many aided tools for preprocessing the data and extracting the important comments such (anxiety, bipolar, and Capture, LIWC (Linguistic Inquiry and Word Count)). They obtained a 54.77% accuracy of depressive indicative in case of user communication with in time range of (midnight-midday) and 45.22% in a reversed time range of communication (midday-midnight).

Authors^18^, developed a model based on data from different social media resources, and then classified users based on their mental health scale and depression. The posts were collected from three sites (Facebook, Live Journal, and Twitter) and they used SVM and Naïve Bayes classifiers for building their model. The model included multi-methods for testing and evaluation of the classifiers. They report accuracy of (57%) using SVM and higher accuracy of (63%) using naïve Bayes. In addition, the paper^19^ hybrid (linear SVM classifier and TF-IDF) and constructed two classes identification problem. For model construction, they used tweets and some textual data, then they evaluated their model using three-word embedding models (skip-gram, CBOW, random trainable). Also, the paper^20^ developed classification and regression models to analysis the colloquial features from social contents towards early depression detection. Their results were enhanced expressively by considering participants variations and extent monitoring time range.

On the other hand, and after its success in computer vision, deep learning is recently employed for text classification, exactly in sentiment analysis. Convolutional Neural Network (CNN) is the most popular technique, and its recitals on text classification are also achieved in the last 5 years compared to the traditional Natural Language Processing (NLP)^21–23^. Paper in^19^ enhanced their models by adapting CNN and RNN techniques. Each model was followed by a fully connected layer (vanilla layer) of 250 hidden units. The author used the Rectified Linear Unit (RELU) activation. They mentioned that CNN enhanced results by an increase of 9% in accuracy on “the Psych 2015 dataset” using 5-fold cross validation. Authors^24^ scrutinized three techniques for gauging the intensity of depression that are: (1) building analysis model based on a 3D facial discriminative features, (2) building a second model based on the language spoken features of the participants and (3) They compared their sentence planted CNN model^14^ with the literature, however the data was gathered from human-to-computer forms, which cause deficiency in the precision of results. The authors also planned to consider additional sorting method based on the sum of depression signs from several previous periods of the same participants. In paper^25^ they utilized a deep-learning model to investigate the posts on social media. They achieved 72% of accuracy and stated that this may improve by focusing on additional features like companionship socially with friends, comments, replies, etc.

As mentioned in the previous works, nearly all the deep learning techniques applied on text have been oriented to the use of word-embedding representation which is recently used in NLP and can be applied using different algorithms such as Word2Vec and GloVe^26^. The main contribution of this proposed work: the authors present an efficient method that uses word embedding and a set of features extracted from Twitter in order to use them in training and classification phases to predict depression, hence acting like pre-protective handling in such healthcare issue. In addition to a comparative analysis with related work towards optimal identification of early signs and symptoms of depression.

**Figure 1:**
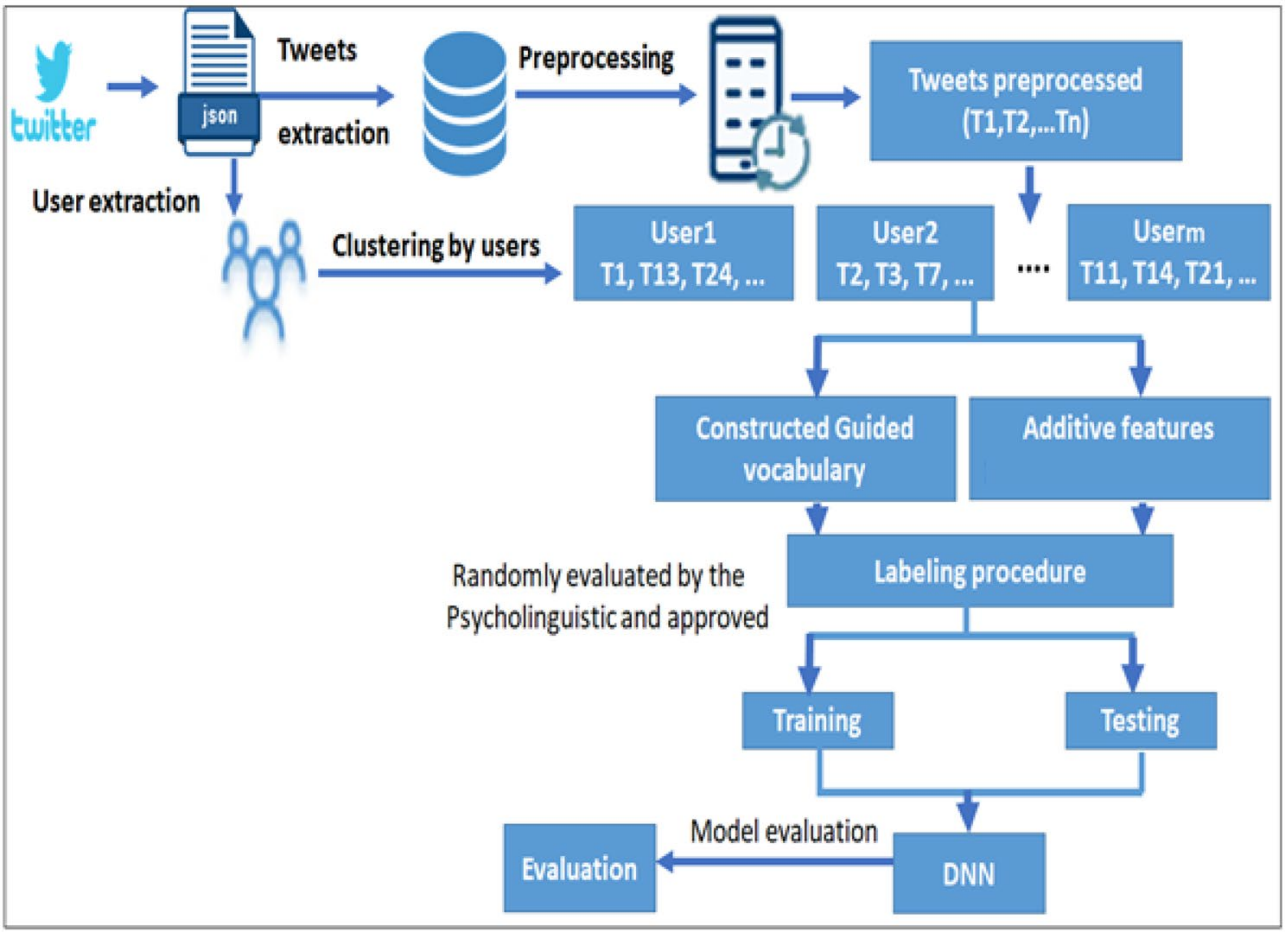
The proposed deep soft computing framework.

**Figure 2:**
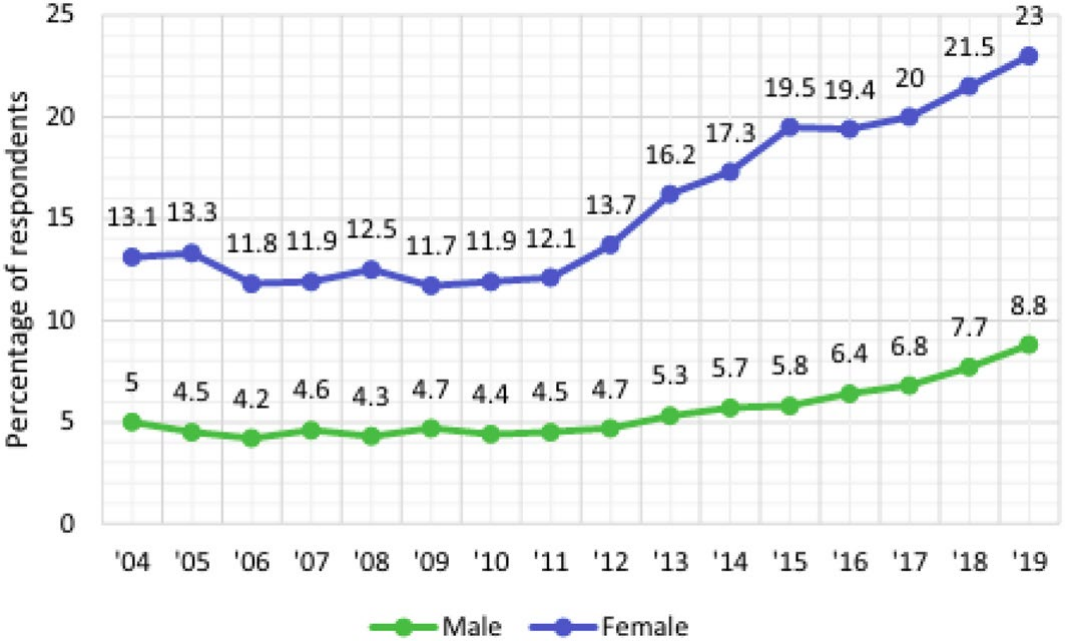
The gender based rates of US youths with depression identification^27,28^.

**Figure 3:**
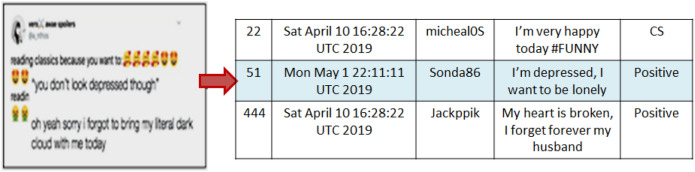
Dataset construction process.

**Table 1:** Sample of the training set.

Id	Date	Username	Description	Status
22	Sat April 10 16:28:22 UTC 2019	micheal05	I’m very happy today #FUNNY	Negative
51	Mon May 1 22:11:11 UTC 2019	Sonda86	I’m depressed, I want to be lonely,	Positive
44	Sat April 10 16:28:22 UTC 2019	JackppiK	my heart is broken, I forget forever my husband	Positive
31	Thu April 20 23:10:18 UTC 2019	micheal05	I want to fly with my love @kiyt	Negative

## Applied method and detailed pre-processing

The proposed approach, displayed in Fig. 1, presents a new method to identify the users of Twitter having the risk of depression from their posts. Beginning from data acquisition to model evaluation; several steps are clarified below:

- Data extraction: in this phase, a list of users and their corresponding posts from Twitter were extracted. The number of followers was selected as a pre-data extraction to limit the scope of the interesting female data set.
- Pre-Processing: the main function of our proposed model is to predict the sentiment of textual tweets as either positive or negative signs of depression; and off course predictions of new textual tweets require applying identical data preparation of the training data. In the experimental results of this paper, we implemented normalization and tokenization of data to clean the tweets textual information, and applied lemmatization to determinate the base form of tokens. For example: list tokens, eliminate all punctuation, discards all words with special characters or of length<= 1 character.
- Feature extraction: first we applied word embedding to construct a guided vocabulary. Then we considered feature representing details about users and their tweets and combined these features for labeling process.
- Word embedding: it is implemented such that similar meanings words have a similar vector representation. In more details, during extraction of tweets, we get the list of words, but it is important to narrow this list to the predictive set targeting a reduction in dimensionality. Additional reduction issue relates the number of trial hypotheses to reach a useful vocabulary which consumes effort of illustration before decision and selection of the reduced set. This reduction in fact needs a guiding role of psycholinguistics and it is achieved in this study. The psycholinguistic participate to save effort with multiple trials and strengthens the scientific approval. Examples of the guided vocabulary by the psycholinguistic: [(‘heartsickness’, 1), (‘despondency’, 2), (‘melancholy’, 3), $\ldots$.] Now each tweet is converted to a list of vocabulary with additive features: For example [(‘heartsickness’, 1, F1, F3 $\ldots$, F17), $\ldots$]. Based on the previous; the tweets are labeled as positive or negative. Random samples of the data were evaluated by the psycholinguistic to approve the procedure.
- Model Creation (DNN): Here, the labeled data from previous phase supplied to the DNN model.
- Model evaluation: The measures [precision, accuracy, recall, and F1 score] are used.

**Figure 4:**
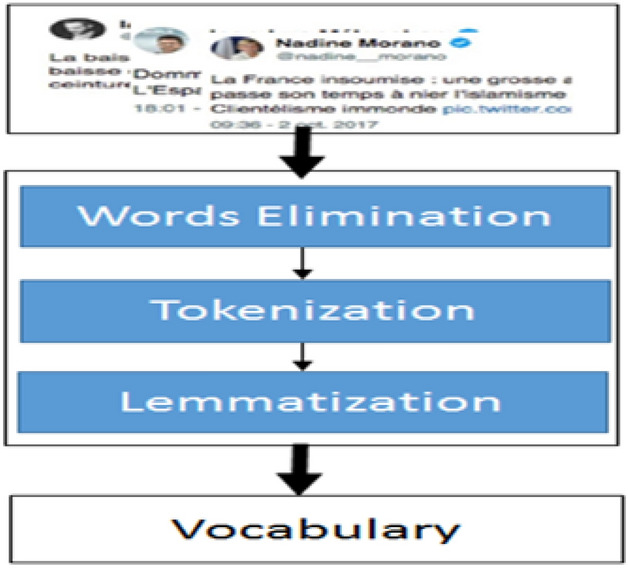
Pre-processing phase.

**Figure 5:**
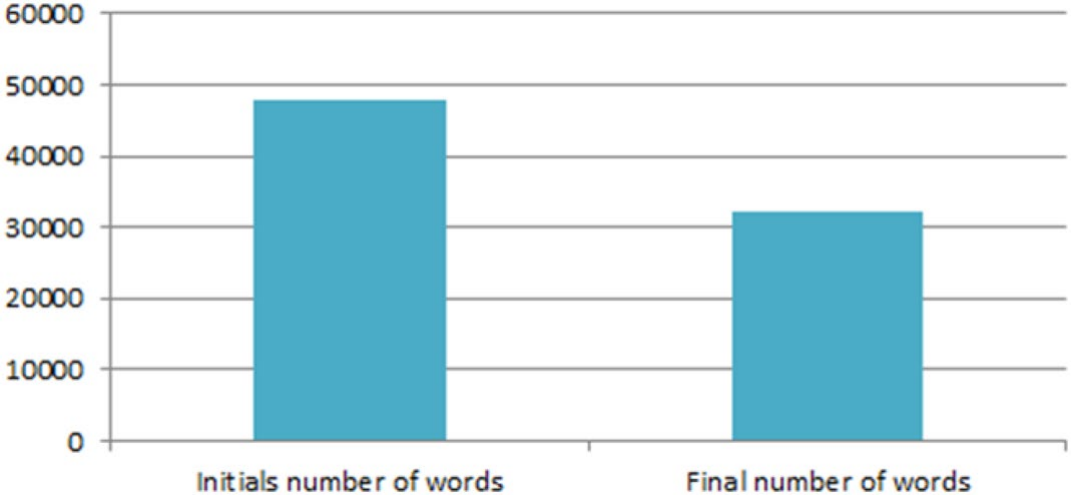
Pre-processing impact on the number of words after eliminating noise.

### Dataset acquisition

Depression is represented by several symptoms and can be detected by user posts and/or tweets. Figure 2, reports the higher rate of women depression than male depression^27^. In the present paper, as explained previously tweets are a good indicator sources for such motivated task, accordingly tweets for women are collected to detect depressive behavior. Specifically and in data preparation, we use the women’s tweets and we eliminate the Retweets.

- The first dataset (General_Data) :

It is collected from seven hundreds participants each of which almost 19 to 22 tweets using Twitter API. We used (10150) for training and (4350) for testing. Figure 3 shows the Dataset construction process. According to authors in paper^8^, expressive words are a good way to predict a user’s personality. Hence, we used the frequency of tweets in both categories of signs and symptom (positive and negative). The indication of depression is (positive) and otherwise is (negative). Table 1 displays some examples of the training set, where the structure of each tweet is represented as the following:

1. Id (e.g., 22).
2. Date of the tweet (e.g, Sat April 10 16:28:2 UTC 2019).
3. User name (e.g, micheal05).
4. Description (e.g, “I’m very happy today”).
5. Status (Depressive state or Control State).

- The second dataset (KSA_Data) is a 2048 tweets gathered from a small sample of Saudi Arabia women accounts [age: range of 17 to 40] and
- The Third dataset (the benchmark ‘CLPsych 2015’ data)^9^: for ease of comparative analysis. The data mainly provide 1,145,000 users labeled as one of three classes [Control, Depressed, and PTSD]. The Control, depressed and PTSD users# are 572,327 and 246; respectively. It provides the gender and age of each user which facilitates the extraction of interest females based sub-samples. From the first class, the females users are almost 74% (423 user). The second class the females users are 80% (261 user). Considering the first two classes of the data set (controlled and depressed), 899 users are obtained. Then with the previous females’ users focusing, we obtained 684 users. The number of tweets is further huge (925,448 and 594,234, for the control and depressed classes, respectively). Hence, and due to resource limitation, we select random sample for training and testing of percentage (19% depressed and 15% non-depressed(control)), (9% depressed data and 9% control),respectively. The rest were eliminated [the genuine statement of diagnosis was found, to prevent any artifact or bias created from our data sampling technique]. This formulated a total of 45390 tweets.

The three used datasets are described in Tables 2 and 3; respectively; in details of training and testing samples, depressed and none depressed samples and the number of female users.

**Table 2:** Tweets’ distribution selected in experiments for three datasets.

Dataset	# of tweets	Training			Testing		
		Total	+	−	Total	+	−
General-Data	14,500	10,150	7028	3122	4350	2734	1616
KSA-Data	2048	1434	1099	335	614	470	144
CLPsych 2015	45,390	31,773	22,530	9243	13,617	9658	3959

**Table 3:** Users’ distribution selected in experiments for three datasets.

Dataset	# of users	Training			Testing		
		Total	+	−	Total	+	−
General-Data	700	495	351	144	205	140	65
KSA-Data	80	56	34	22	24	16	8
CLPsych 2015	684	479	269	210	205	144	61

### Pre-processing

Preprocessing of data rapid improvements of models results and time of training especially in text classification, which guide for relevant embedding words (see Fig. 4). The stopping words such [the, that, we, are, etc.,] were eliminated during tokenization, as these are not-informative tokens. The removing of the inflectional ending of words and conversion to their base form, are executed in the lemmatization. In the morphological phase, tokens are converted to its stem form by elimination prefixes or suffixes or plural addition (eg. happy, un-happy-ness). Figure 5 displays the pre-processing effect on the collected dataset before and after removing noise suppression [e.g.: Icons, Hashtags, Links, Gif images, @mentions, spatial characters]. The figure shows a reduction of the vocabulary set by 35% of its size from the original corpus.

**Figure 6:**
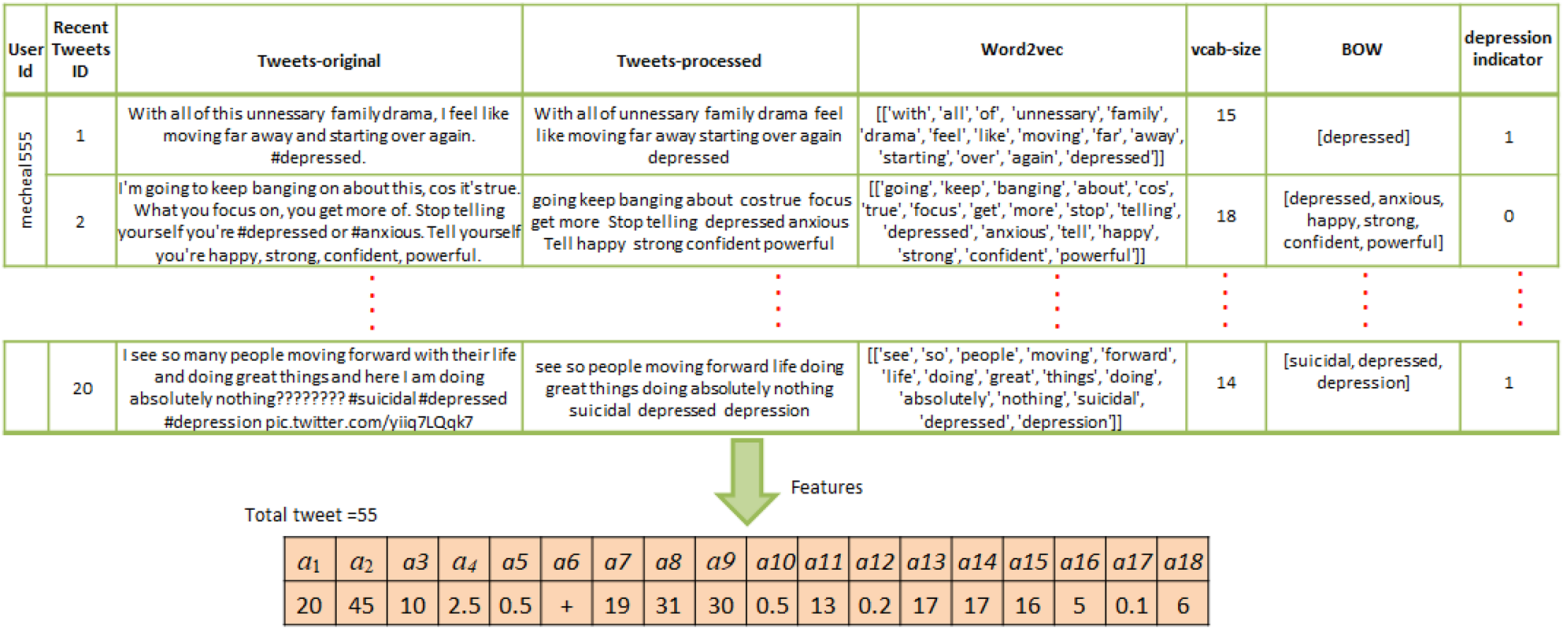
Feature extraction example for single user.

**Figure 7:**
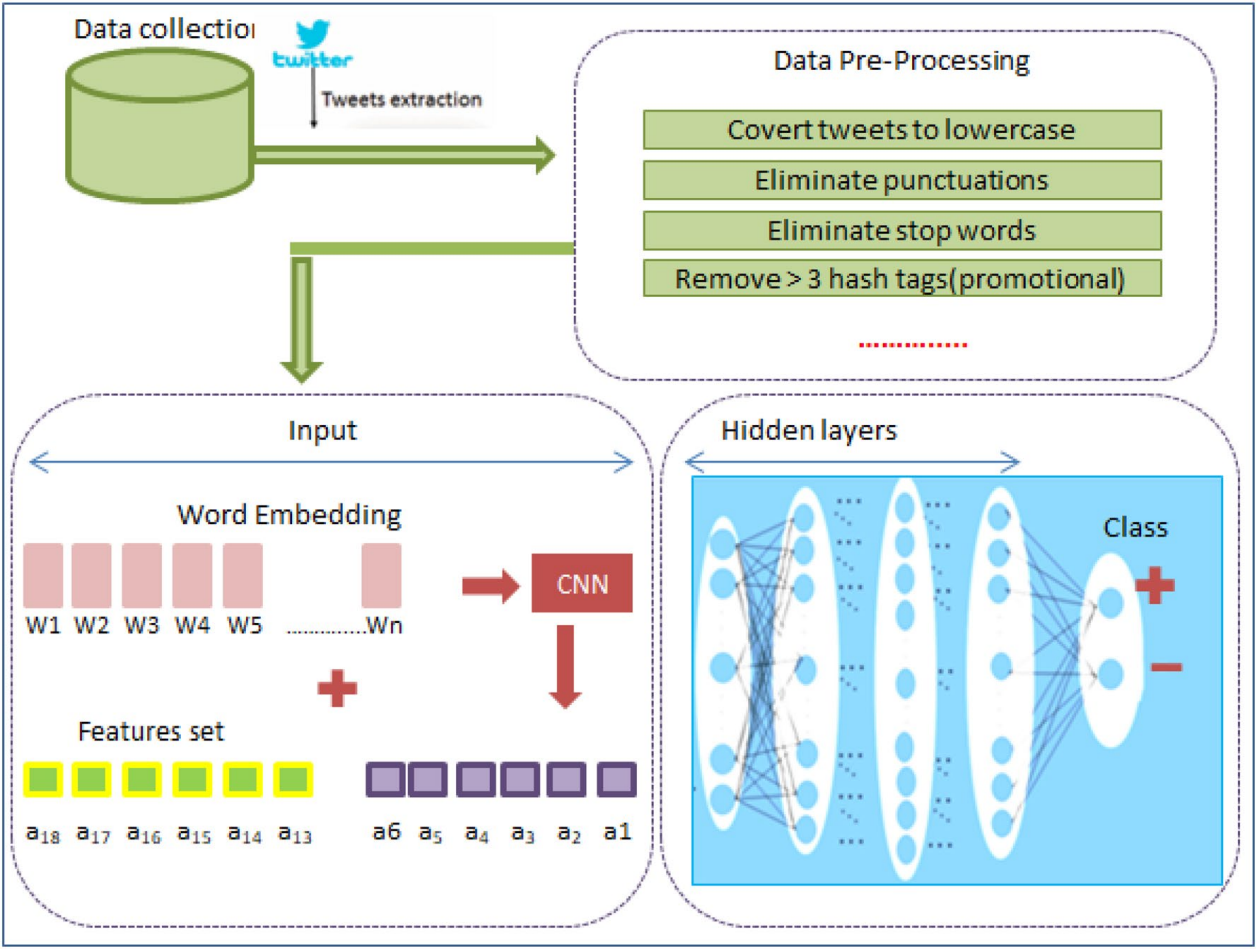
The constructed deep soft-computing model.

### Selecting features

In this section, the details of selecting the discriminative features used in training are presented. The 17 features are labeled from $\hbox {a}_{{1}}$ to $\hbox {a}_{{17}}$. Two main considerations of feature extraction were necessary. For ease of illustration, we categorized both in two separate lists before and after pre-processing the data, labeled $\hbox {set}_{(1)}$ and $\hbox {set}_{(2)}$. $\hbox {set}_{(1)}$= {$\hbox {a}_{{7}}$, $\hbox {a}_{{8}}$, $\hbox {a}_{{9}}$, $\hbox {a}_{{10}}$, $\hbox {a}_{{11}}$, $\hbox {a}_{{12}}$, $\hbox {a}_{{13}}$, $\hbox {a}_{{16}}$, $\hbox {a}_{{17}}$}, and $\hbox {set}_{(2)}$= {$\hbox {a}_{{2}}$, $\hbox {a}_{{3}}$, $\hbox {a}_{{4}}$, $\hbox {a}_{{5}}$, $\hbox {a}_{{6}}$, $\hbox {a}_{{14}}$, $\hbox {a}_{{15}}$, $\hbox {a}_{{18}}$}, respectively. Both sets are valuable for narrowing the collected data and guide building the right model. $\hbox {Set}_{(1)}$ is important for user description, while $\hbox {set}_{(2)}$ is important for tweet description. $\hbox {a}_{{1}}$ is # of recent tweets. $\hbox {a}_{{2}}$ and $\hbox {a}_{{3}}$ are # of depressed, non-depressed tweets, respectively, while $\hbox {a}_{{4}}$ and $\hbox {a}_{{5}}$ are # of recent tweets in both categories, respectively. $\hbox {a}_{{6}}$ is the label of user status. $\hbox {a}_{{7}}$ and $\hbox {a}_{{8}}$ are # of followers and Age, respectively. The # of Hashtags and its average value, mentions and its average are $\hbox {a}_{{9}}$, $\hbox {a}_{{10}}$‏, $\hbox {a}_{{11}}$, $\hbox {a}_{{12}}$, respectively. $\hbox {a}_{{13}}$, $\hbox {a}_{{14}}$, and $\hbox {a}_{{15}}$ are retweets#, words/tweet# and their average, respectively. $\hbox {a}_{{16}}$ and $\hbox {a}_{{17}}$ are # of URL and their average. Finally, $\hbox {a}_{{18}}$ is the frequency of guided words of the psychologist.

In fact, the number of tweets ($\hbox {a}_{{1}}$) corresponds to the last participant tweets. Due to the huge tweets, we considered a predefined X most recent for dimensionality reduction reason. a1 can be less than X if the user has a new Twitter account or he doesn’t use Twitter frequently. X is determined based on the period where we need to see the user status, see Eq. (1). Figure 6 for illustration of extraction of features.

1 $$\begin{aligned} X = {\left\{ \begin{array}{ll} 10 &{} \quad \text {if }Period \, =1\, Week\\ 20 &{} \quad \text {if } Period \,=1 \, Month \end{array}\right. } \end{aligned}$$

$\hbox {a}_{{2}}$ and $\hbox {a}_{{3}}$ are categorized using the following proposition: If the tweet ($\hbox {T}_{{i}}$) is depressed, its state (S($\hbox {T}_{{i}}$)) is positive otherwise it is negative. Let n is the number of users and list of USERS =[$\hbox {U}_{{1}}$, $\hbox {U}_{{2}}$ $\ldots$ $\hbox {U}_{{n}}$]. Then for $\hbox {U}_{{k}}$, the early sign and symptoms diagnosis is calculated in Eq. (2).

2 $$\begin{aligned} a_6=Status(U_k) = {\left\{ \begin{array}{ll} + &{} \quad \text {if } a_2 \text { >} a_3 \\ - &{} \quad \text {Otherwise } \end{array}\right. } \end{aligned}$$

where

3 $$\begin{aligned} {\left\{ \begin{array}{ll} a_2 = \sum _{i-1}^{a_1}{ (S(T_i) =+ ) }\\ a_3 = \sum _{i-1}^{a_1}{ (S(T_i) =- ) } \end{array}\right. } \end{aligned}$$

and

4 $$\begin{aligned} {\left\{ \begin{array}{ll} a_4 = \frac{a_2}{a_1}\\ a_5 = \frac{a_3}{a_1} \end{array}\right. } \end{aligned}$$

In section “Related work”, authors in^4,29,30^ believed that $\hbox {a}_{{7}}$; the number of followers; reflect a high valuable indicator for the degree of the sociality of participants. High sociality is connected to large number of followers and vice versa. This indicates signs of depression with low degree of sociality especially in young ages. Therefore $\hbox {a}_{{7}}$ and $\hbox {a}_{{8}}$ are important in building the presented approach.$\hbox {a}_{{9}}$, $\hbox {a}_{{11}}$, and $\hbox {a}_{{13}}$ represent the consequences of account’s activities on depression detection. Mainly hashtags mentions, and retweets are a very good indicators during the analysis^30,31^.

in the other hand, the Bag of Words (BOW)^32–34^ is a model used in language processing, which keeps a vector of words having the size of vocabulary for each document (tweet). If the vocabulary size is important, the training and classification tasks will be expensive and hard to do. One solution for that is the feature extraction that selects a specific set of words (named embedding words) from the training set and uses it for the classification task. To construct the embedding words vector, we trained Word2Vec of two layers to identify a word (or predefined list of words) having similar context. Two directions are used (CBOW (Continuous Bag-Of-Words) and Skip-gram)^32–34^. The first predicts one word from context, while the second predicts the context from a given word. The intention of using both are tweets reduction that corresponding a reduction in vocabulary.

### Implementation

The model in Fig. 7 is implemented as follow: each layer receives input from the preceding layer and forward outputs to the next layer. All are fully connected. The figure shows initial input is a set of tweets features plus the word. The output layer consist of a binary representation of (+/or −). The standard back-propagation method is applied for the correction of error, while the Sigmoid (Eq. 5) is used as an activation functions during training and Softmax function (Eq. 6) to produce the final classification.

5 $$\begin{aligned} f(x)= & {} \frac{1}{(1+e^{-x})} \in (0,1) \end{aligned}$$

6 $$\begin{aligned} Softmax(P,L)= & {} \frac{exp(v)}{\sum _{i=1}^{L}{ ( exp(v^\wedge ) } } \end{aligned}$$

To develop the proposed DNN, we used the keras deep learning library, and adopted the framework according the following: we set the number of hidden layers = 4; used dropout = 0.5 as regularization technique; stop the training when number of epochs = 200 with batch size = 256; the Gaussian distribution with a standard deviation = 0.01 for obtaining the weights and biases initialization; used the Softmax as a Los function and Sigmoid as an activation functions.

**Table 4:** Clarification of the evaluation parameters in this presentation

Parameter	Meaning	Clarification
$t_p$	True positives	The # of + tweets and predicted +
$f_p$	False positives	The # of − tweets and predicted +
$t_n$	True negatives	The # of − tweets and predicted −
$f_n$	False negative	The # of + tweets and predicted −

**Figure 8:**
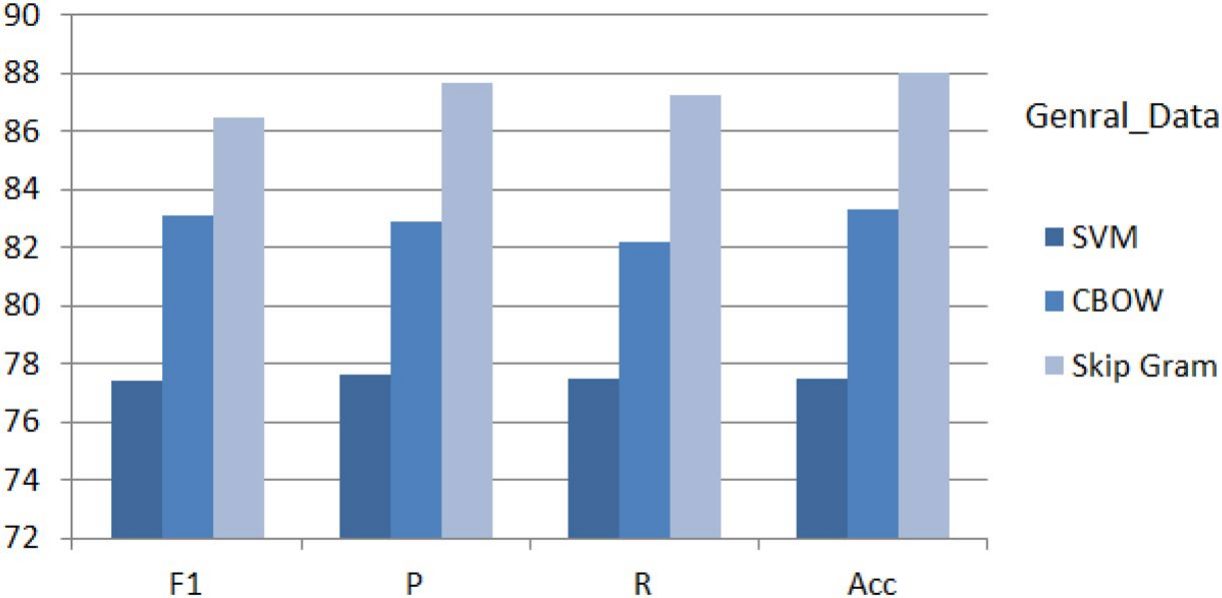
Implementation of SVM, CBOW and Skip Gram on ‘General_Data’.

**Figure 9:**
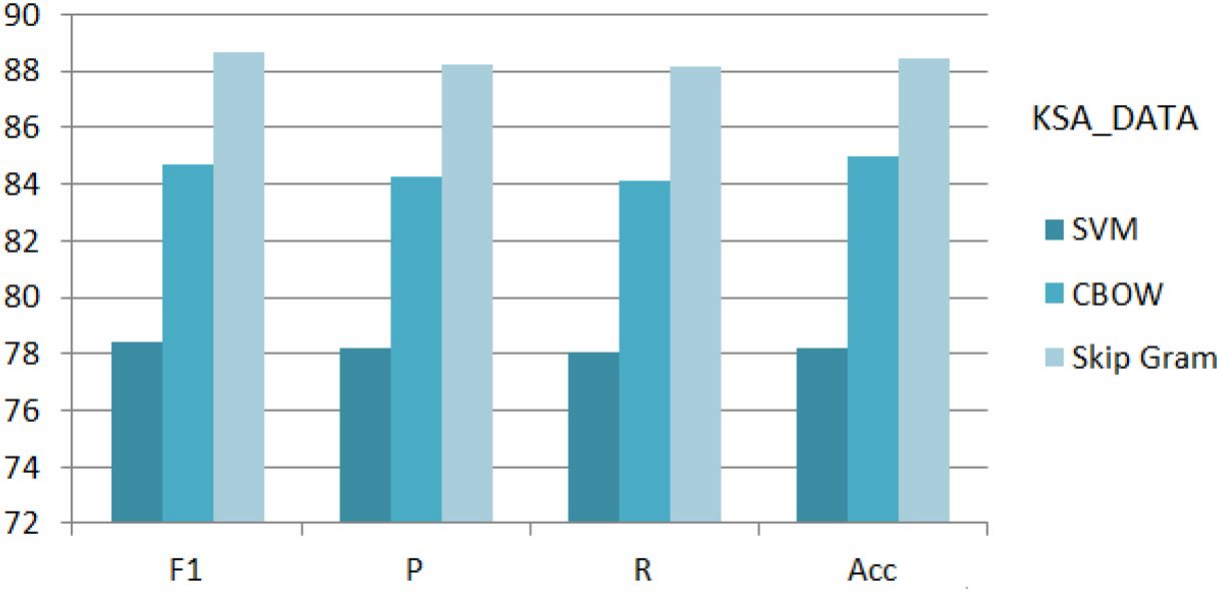
Implementation of SVM, CBOW and Skip Gram on ‘KSA_Data’.

**Figure 10:**
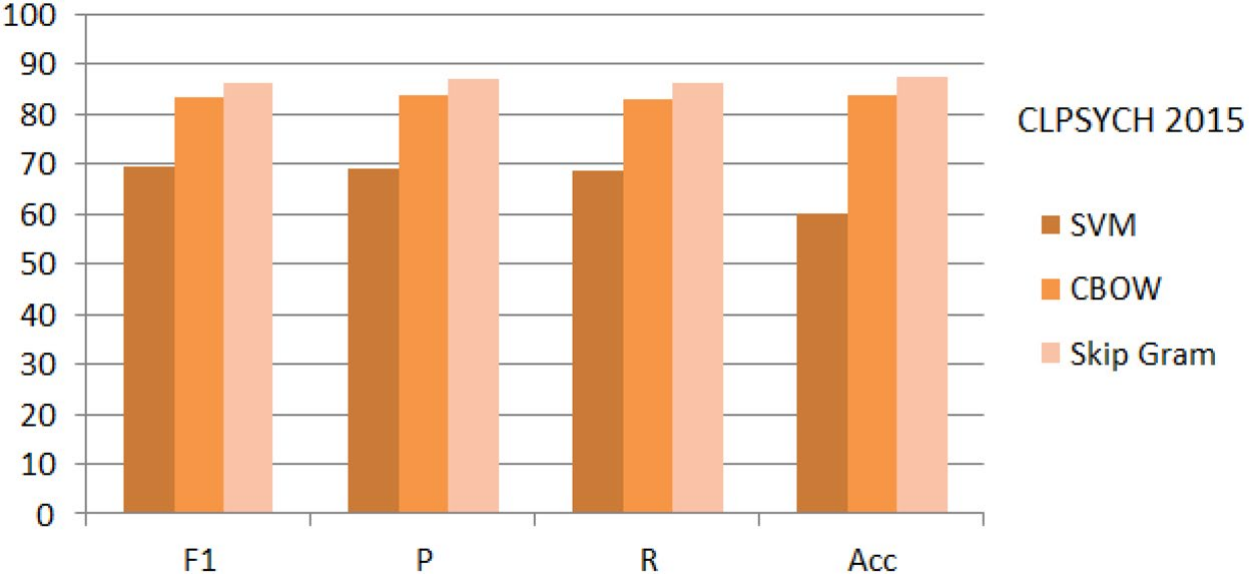
Implementation of SVM, CBOW and Skip Gram on ‘CLPSYCH 2015’.

## Experimental and discussion

Implementing the proposed architecture is followed with performance analysis and evaluation on collected three data sets described in data accusation section in details. In addition to literature benchmark comparative analysis verses variant models for the third dataset.Four methods were used for evaluation of performance as follow: Accuracy (Ac, Eq. 7), Precision (Pr, Eq.  8), recall (Re, Eq. 9), and F1-score (F1, Eq. 10)^1–3,22^. Table 4 describes briefly tp, fp, tb and fn.

7 $$\begin{aligned} Acc= & {} \frac{(t_p+t_n)}{(t_p+t_n+f_p+f_n)} \end{aligned}$$

8 $$\begin{aligned} P_r= & {} \frac{(t_p+t_n)}{(t_p+f_p)} \end{aligned}$$

9 $$\begin{aligned} R_e= & {} \frac{(t_p+t_n)}{(t_p+f_n)} \end{aligned}$$

10 $$\begin{aligned} f_1= & {} \frac{2\times P_r\times R_e}{(P_r+R_e)} \end{aligned}$$

**Figure 11:**
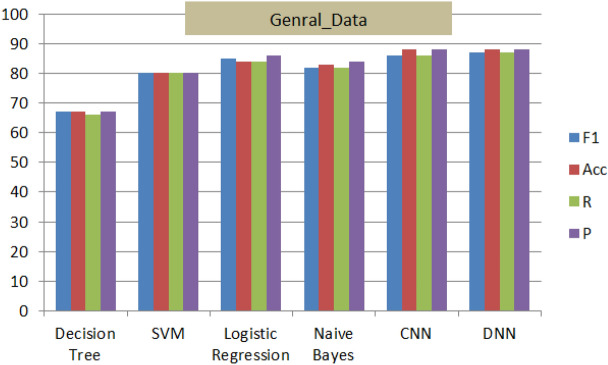
Performance of M(7) combined with different shallow and deep techniques for the General-Data.

**Figure 12:**
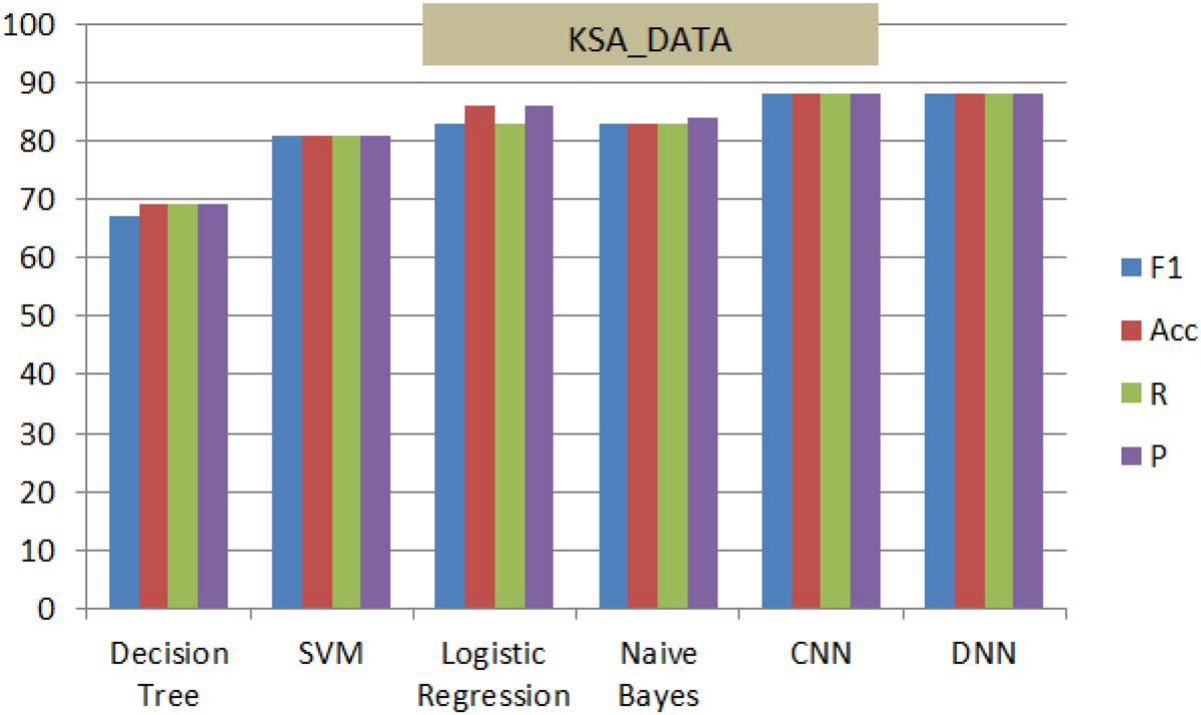
Performance of M(7) combined with different shallow and deep techniques for the ‘KSA_Data’.

**Figure 13:**
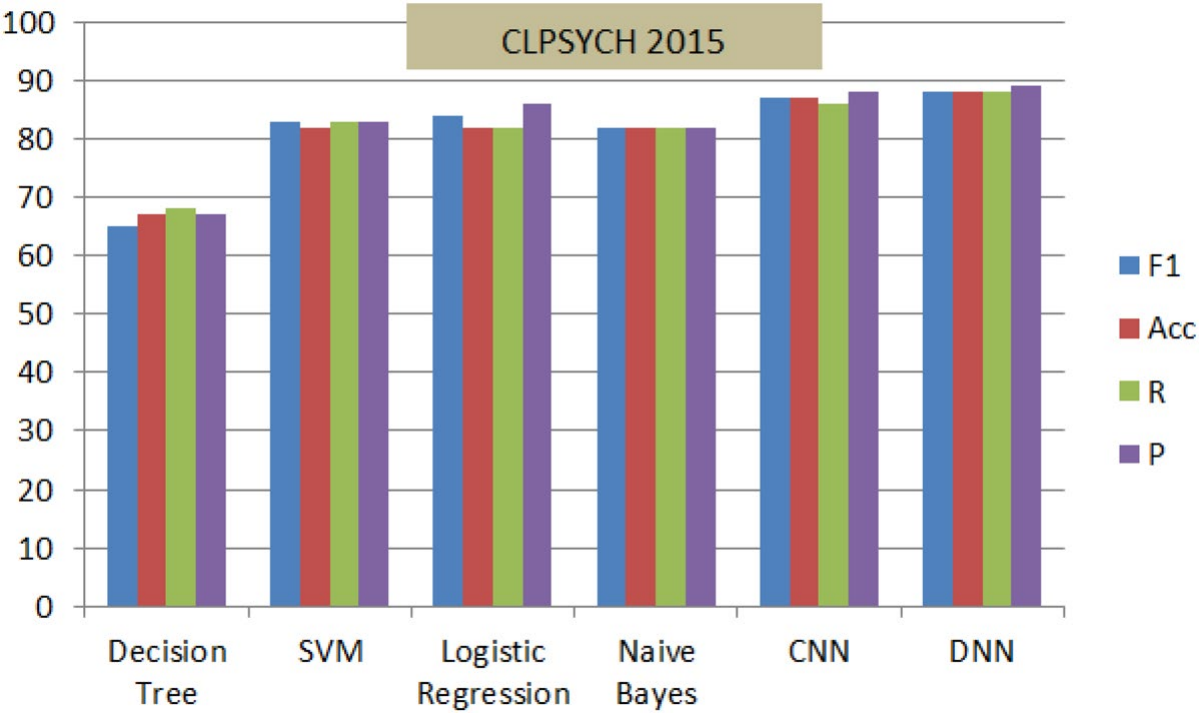
Performance of M(7) combined with different shallow and deep techniques for the ‘CLPSYCH 2015’.

**Table 5:** The proposed models for comparison.

Model	Description
M(1)	Word embedding
M(2)	$a_1$ to $a_6$
M(3)	$a_1$ to $a_6$ and Word embedding
M(4)	$a_7$ to $a_17$
M(5)	$a_7$ to $a_17$ and Word embedding
M(6)	$a_1$ to $a_17$
M(7)	$a_1$ to $a_17$ and Word embedding

**Table 6:** Precision of our models on the General-Data dataset with 10-fold cross-validation.

	M(1)	M(2)	M(3)	M(4)	M(5)	M(6)	M(7)
Decision tree	65.32	65.39	66.50	65.40	67.60	67.75	67.75
SVM	77.10	77.70	79.58	78.51	80.11	80.23	80.62
Logistic regression	83.44	84.10	84.98	84.45	85.32	85.65	86.01
Naive Bayes	82.51	82.50	82.87	82.52	83.22	83.66	84.54
CNN	87.32	87.32	87.53	88.32	87.66	88.07	88.29
DNN	86.22	86.13	88.11	86.45	88.12	87.45	88.46

**Table 7:** Comparative analysis for CLPSYCH 2015 dataset

Authors	Model	P	R	F1	Acc
Orabi et al.^19^	CNN	87.4	87.02	86.9	87.9
Orabi et al.^19^	MultichannelPoolingCNN	87.2	86.6	86.4	87.5
Shweta et al.^39^	BERT FiLaMTL-fine-tuned	76.46	73.65	75.03	70.7
Proposed model	DNN based model	82.1	87.87	87.3	89

**Table 8:** Comparative analysis for CLPSYCH 2015 dataset including user and tweets statistics (D: Depressed, C: Control)

Authors	# Users/train		# Users/test		# Tweets/train		# Tweets/test		P	R	F1
	D	C	D	C	D	C	D	C			
Shweta et al.^39^	69	151	150	300	110,585	254,786	284,777	541,282	76.46	73.65	75.03
our DNN model	269	210	144	61	22,530	9243	9658	3959	88.1	87.8	87.3

Among the Word2Vec extraction techniques^22,31,33^, the CBOW and Skip-gram reported an impressive performance recently^4,5,27^. Accordingly, both techniques are implemented on the three datasets. Figures 8, 9, 10 show the evaluation of the proposed deep architecture that recalled two word embedding for training (CBOW and the Skip Gram) in addition to the SVM as a baseline. From the figures, and for the three sets, the proposed DNN deep architecture obtained reported higher (Accuracy, Precision, recall, and F1-score) results than the SVM classifier. In addition, skip Gram showed higher results than CBOW. After near 60 epochs the network is stabilized for SVM, and after 100 epochs the network is stabilized for proposed architecture. Regarding the General_Data, we obtained for the depressed case an F1-score equal to 77.8%, 83.11%, and 86.44% respectively by applying SVM, CBOW, and Skip Gram. According to these results, DNN is nominated as a classifier in text classification and its strong impact on depression detection.

Cross-validation is adopted to evaluate the proposed model. Results in this section are reported using 10-fold cross-validation, which are carried out by partitioning the owner dataset (Genera_Data) into 10 folds where 9 are used for training and one for testing, then this procedure is repeated 10 times averaging results. In the following a comparative analysis of two implemented deep learning models; (1) Basic CNN holds identical configuration in^34^ and (2) our proposed DNN architecture; with traditional techniques (Decision tree^35,36^, SVM^17^, Logistic Regression^37^ and Naïve Bayes^38^) is presented. Also each approach is analyzed from variant permutation of features, we name as models (M(1) to M(2)) to check optimal findings. In fact, these models depend from the set of features and the words embedding, extracted from our datasets.

Table 5, present description of different models we developed for comparisons analysis of the dataset (mainly the first two data sets). Also, we used these model for comparison analysis of the benchmark data (third dataset), because the mentioned previous papers used mostly a different random samples. This leads to experiment comparisons setting with low certainty. On the other hand, the comparison while fixing the data and with variant models validates the results more. Table 6 shows the precision of all classification methods including the proposed approach. Generally, deep learning classifiers (CNN and DNN) keep the best precision in comparison with the traditional machine learning techniques. The DNN method (88.46) reports the higher precision; this result is so close to the reported by the CNN because both methods used identical word embedding set, identical set of features and even same activation function. We can observe also from Table 6 M(7) recorded the highest precision and classification performance. The competitive higher performance of M(3), M(5) and M(7) is believed to be by combing features and word embedding while other models recorded poor performance because they consider dependencies among features and words embedding, which is proved true in this context. All features used in the experiments hold a degree of dependency. Therefore, when all the features are present in the same model, here M(7), the efficiency is appreciated.

Figures 11, 12 and 13, display the performance of the proposed method using the three datasets (General_Data, KSA_Data and CLPsych 2015). The optimal performance based on F1-scores, Precision, Recall and accuracy are reported for (CNN and DNN) compared to the other traditional techniques. The DNN-based model; on the three datasets; recorded highest results of average of 88% while Decision Tree^38^ recorded the lowest results of 67%. This nominates the proposed DNN model for similar mining tasks and supports its efficiency and performance.

Tables 7 and 8 show, a comparative analysis for the benchmark CLPSYCH 2015 dataset. In fact, although Table 7 shows the higher reported results of the proposed model in this study using the standard evaluation criteria, the authors of this study rather prefer comparing results such exist in Table 6. This is because, analysis in Table 8 proves that these comparisons may be unfair when considering the selected data set samples. The Table 8 shows different selected samples and sizes as well in each paper. Accordingly, The authors of this paper were committed to select the female subset only, while other papers used the whole dataset or may be different tweet number for each user. Specifictly, not identical data set.  Accordingly, we attempt comparing results in both directions (1) using different models with identical data samples. (2) using different models and different data samples. In both comparison, the proposed model compete efficiently.

## Conclusion and future work

In this study, we introduced a new deep architecture to extract the early signs and symptoms of the women’s depression from their expressive profiles. The paper considered the most valuable features based on the literature related work presented in section “Related work” from textual information such [of tweets per account, followers, links, hashtag, etc]. We integrated the mentioned features with two word embedding algorithms to build the proposed DNN model for classifying signs extracted as indicator of depression or not. Additionally we permutated features with the word embedding to obtain variant subsets of features and hence variant models architectures [M(1) to M(1)]. This guided us towards discriminative features and optimal performance. Finally we trained by implemented models using three types of dataset: (1) ‘General_data’, contains near 14,500 tweets, (2) ‘KSA_Data’ contains 2048 tweets from 80 women in Saudi Arabia and (3) the benchmark ‘CLPsych 2015’. The computational experiments nominated the Skip Gram word embedding algorithm over CBOW with reported higher performance over the three datasets. Also, it provides a valuable analysis for the proposed DNN architecture in a comparison with the traditional existing machine learning techniques and the basic CNN framework applied for the identical issue. For future directions, some attempts towards attention techniques for the deep models is intended to be applied. Also, variant social applications may be considered for gathering the communication data.
